# Addressing the Challenges of Hepatitis C Cure and Persistent Risk of Hepatocellular Carcinoma

**DOI:** 10.3390/v11050441

**Published:** 2019-05-15

**Authors:** Thomas F. Baumert, Yujin Hoshida

**Affiliations:** 1 Inserm, U1110, Institut de Recherche sur les Maladies Virales et Hépatiques, F-67000 Strasbourg, France; 2 Université de Strasbourg, F-67000 Strasbourg, France; 3 Institut Hospitalo-Universitaire, Pôle Hépato-digestif, Nouvel Hôpital Civil, F-67000 Strasbourg, France; 4 Liver Tumor Translational Research Program, Harold C. Simmons Comprehensive Cancer Center, Division of Digestive and Liver Diseases, University of Texas Southwestern Medical Center, Dallas, TX 75390, USA

Chronic hepatitis C virus (HCV) infection is a major cause of liver disease and hepatocellular carcinoma (HCC)—the second leading, and rising, cause of death due to cancer world-wide. Following the discovery of the virus, just three decades ago, the field has succeeded in developing methods that have changed the safety of blood products, understanding molecular virology, epidemiology, clinical pathogenesis of HCV infection, and unraveling targets for antiviral therapies [1,2]. Most importantly, these discoveries have resulted in the development of safe and highly-effective direct-acting antivirals (DAAs) enabling viral cure in more than 90% of treated patients.

Nevertheless, major clinical and scientific challenges remain: Therapy is still only available to a fraction of infected patients worldwide and many patients remain undiagnosed and/or live in countries where therapy is unattainable. An urgently-needed HCV vaccine to eradicate infection is not yet available. Moreover, despite an efficient viral cure, the risk of developing HCC remains elevated, although substantially reduced, particularly in patients with advanced liver fibrosis [3]. Several earlier studies have suggested evidence for an increased risk of HCC recurrence in patients treated with DAAs, although subsequent studies have shown that clinically-observed effects on HCC incidence is likely comparable between DAAs and the former interferon-based regimens. However, experimental mechanistic studies have suggested that their molecular consequence may be different between the new and old regimens with regard to modulation of host immunity and oncogenic pathways.

In this Special Issue entitled “Cure of Hepatitis C Virus Infection and Hepatocellular Carcinoma”, a panel of leading experts provide an overview of this rapidly evolving field, focusing on the next challenges in viral eradication and HCC prevention in the era of DAA. Pradat et al. summarized the changing landscape of HCV epidemiology as well as currently available evidence and future prospect about HCC incidence after sustained virologic response (SVR) [4]. Sanduzzi-Zamparelli et al. reviewed the latest clinical evidence about post-DAA HCC recurrence, one of the major concerns over the past few years [5]. Alazard-Dany et al. overviewed the latest knowledge about HCV life cycle and new antiviral strategies directed to viral and/or host targets [6]. Virzi et al. summarized cellular signaling pathways modulated by HCV as potential targets for HCC preventive intervention [7]. Luxenburger et al. reviewed changes in T cell response after viral cure, particularly by DAAs, and their involvement in post-SVR pathogenesis [8]. Hayes et al. assembled currently reported experimental data on molecular mechanisms of post-SVR HCC development, which may be different between DAAs and interferon-based regimens [9]. Plissonnier et al. discussed non-coding RNAs for their roles in liver disease pathogenesis and as circulating biomarkers in post-SVR HCC [10]. These rapidly accumulating clinical and experimental findings and ongoing studies will collectively contribute to the eventual elimination of HCV infection and improved clinical management of post-SVR HCC.

## References

[1] Baumert T.F., Schuster C., Cosset F.L., Dubuisson J., Hofmann M., Tautz N., Zeisel M.B., Thimme R. (2016). Addressing the next challenges: A summary of the 22nd international symposium on hepatitis c virus and related viruses. J. Hepatol..

[2] Chung R.T., Baumert T.F. (2014). Curing chronic hepatitis C—the arc of a medical triumph. N. Engl. J. Med..

[3] Baumert T.F., Juhling F., Ono A., Hoshida Y. (2017). Hepatitis C-related hepatocellular carcinoma in the era of new generation antivirals. BMC Med..

[4] Pradat P., Virlogeux V., Trepo E. (2018). Epidemiology and elimination of HCV-related liver disease. Viruses.

[5] Sanduzzi-Zamparelli M., Boix L., Leal C., Reig M. (2019). Hepatocellular carcinoma recurrence in HCV patients treated with direct antiviral agents. Viruses.

[6] Alazard-Dany N., Denolly S., Boson B., Cosset F.L. (2019). Overview of HCV life cycle with a special focus on current and possible future antiviral targets. Viruses.

[7] Virzi A., Roca Suarez A.A., Baumert T.F., Lupberger J. (2018). Oncogenic signaling induced by HCV infection. Viruses.

[8] Luxenburger H., Neumann-Haefelin C., Thimme R., Boettler T. (2018). HCV-specific T cell responses during and after chronic hcv infection. Viruses.

[9] Hayes C.N., Zhang P., Zhang Y., Chayama K. (2018). Molecular mechanisms of hepatocarcinogenesis following sustained virological response in patients with chronic hepatitis C virus infection. Viruses.

[10] Plissonnier M.L., Herzog K., Levrero M., Zeisel M.B. (2018). Non-coding RNAs and hepatitis C virus-induced hepatocellular carcinoma. Viruses.

